# Affinity to cellulose is a shared property among coiled-coil domains of intermediate filaments and prokaryotic intermediate filament-like proteins

**DOI:** 10.1038/s41598-018-34886-7

**Published:** 2018-11-08

**Authors:** Niklas Söderholm, Ala Javadi, Isabel Sierra Flores, Klas Flärdh, Linda Sandblad

**Affiliations:** 1 Department of Molecular Biology, Umeå University, 901 87 Umeå, Sweden; 2 Department of Biology, Lund University, 22362 Lund, Sweden

## Abstract

Coiled-coil domains of intermediate filaments (IF) and prokaryotic IF-like proteins enable oligomerisation and filamentation, and no additional function is ascribed to these coiled-coil domains. However, an IF-like protein from *Streptomyces reticuli* was reported to display cellulose affinity. We demonstrate that cellulose affinity is an intrinsic property of the IF-like proteins FilP and Scy and the coiled-coil protein DivIVA from the genus *Streptomyces*. Furthermore, IF-like proteins and DivIVA from other prokaryotic species and metazoan IF display cellulose affinity despite having little sequence homology. Cellulose affinity-based purification is utilised to isolate native FilP protein from the whole cell lysate of *S*. *coelicolor*. Moreover, cellulose affinity allowed for the isolation of IF and IF-like protein from the whole cell lysate of *C*. *crescentus* and a mouse macrophage cell line. The binding to cellulose is mediated by certain combinations of coiled-coil domains, as demornstrated for FilP and lamin. Fusions of target proteins to cellulose-binding coiled-coil domains allowed for cellulose-based protein purification. The data presented show that cellulose affinity is a novel function of certain coiled-coil domains of IF and IF-like proteins from evolutionary diverse species.

## Introduction

Numerous species of prokaryotic and eukaryotic origin are predicted to encode coiled-coil intermediate filament (IF) or IF-like proteins^1–4^. However, the first IF to be studied was of metazoan origin, and IF proteins came to be defined by their distinct tripartite domain organisation and capability to self-assemble into non-poplar filaments independent of cofactors. The tripartite organisation of IF proteins are comprised of globular non-alpha-helical head and tail domains enclosing a central α-helical region that consists of coiled-coil domains^5^. IF-like proteins share the tripartite domain organisation that defines metazoan IF proteins but display high sequence variability and diverse cellular functionality.

Studies on bacterial species including *Helicobacter pylori*, *Caulobacter crescentus*, and *Streptomyces coelicolor* show that their IF-like proteins are capable of self-assembly into filaments *in vitro* and are important for bacterial morphology^1,6–13^. The genome of *S*. *coelicolor* codes for 7,825 proteins, of which only FilP and Scy have been reported as IF-like proteins. Together with the coiled-coil domain containing protein DivIVA, Scy is part of the tip-located polarisome, a multiprotein complex that is responsible for the apical growth of *Streptomyces* hyphae^11,14^. FilP is localised immediately adjacent to the polarisome^10^. Bioinformatic analysis shows that FilP and Scy have a similar domain organisation and that DivIVA shares some of their sequence characteristics^15^. Bacterial two-hybrid systems, pelleting assays, and pulldowns have shown that FilP, Scy and DivIVA are interacting^10,11^. FilP is subjected to post-translational modifications such as phosphorylation and N-acetylation^16,17^. However, most *in vitro* studies of FilP filament formation and other IF-like proteins have so far been performed using recombinant proteins^1,6,10,11,13^.

Before FilP was considered as an IF-like protein, a FilP orthologue from *Streptomyces reticuli* denoted AbpS was discovered to display high affinity to Avicel during a screen for cellulose binding proteins^18^. Avicel is microcrystalline cellulose prepared from hydrolysed cellulose pulp. The amorphous fraction of cellulose is to a large extent removed during the hydrolysis leading to enrichment of the microcrystalline cellulose. *S*. *reticuli* belongs to a group of species with the ability to utilise crystalline cellulose as their sole source of carbon. It was therefore hypothesised that the FilP orthologue AbpS was involved in the metabolism of crystalline cellulose. However, FilP is a part of the *Streptomyces* core genome and is both found in species that can and cannot metabolise crystalline cellulose^19^. This suggests that the cellulose affinity observed for the FilP orthologue AbpS has additional or alternative roles to being involved in cellulose metabolism.

*Streptomyces* species synthesize a cellulose-like β-(1-4) glucan located at the hyphal tip, which forms fimbriae that enable attachment to surfaces. The cellulose-like molecule is produced by a tip-localised cellulose synthase-like protein encoded by the *cslA* gene^20,21^. It has been proposed that the cellulose affinity observed for the FilP orthologue AbpS could anchor the cellulose-like molecules to the surface of the bacteria^21^. The Carbohydrate-Active enzymes Database (CAZy, http://www.cazy.org/) has listed 265 proteins from *S*. *coelicolor* that contain carbohydrate binding modules (CBMs), which are potentially involved in the binding and degradation of polysaccharides and sugars^22^. The strict definition of a CBM postulates that it should exist as a module within a carbohydrate active enzyme. This separates CBMs from non-catalytic carbohydrate binding proteins, such as lectins and sugar transport proteins, which do not have to have enzymatic activity. Neither FilP, Scy nor DivIVA orthologues in *Streptomyces* contain any described CBM, and no cellulase activity was detected for AbpS^18^.

In this study, we show that IF-like proteins and DivIVA from diverse bacterial species and metazoan intermediate filaments proteins display high affinity to cellulose. Certain coiled-coil domains are shown to be responsible for the interaction. The cellulose specific interaction described here is used to isolate natively expressed coiled-coil proteins and recombinantly expressed fusion proteins.

## Results

### FilP, Scy and DivIVA from *Streptomyces spp*. display specific cellulose affinity

**Figure 1:**
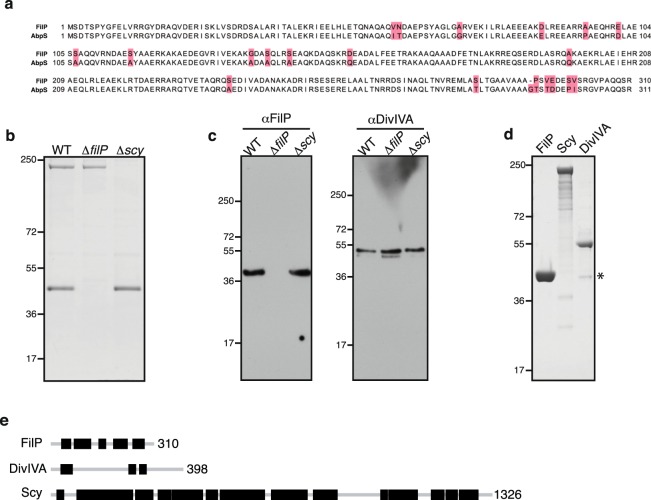
Cellulose binding proteins from *S*. *coelicolor*. (**a**) Clustal Omega alignment of *S*. *reticuli* AbpS and *S*. *coelicolor* FilP. Red boxes indicate mismatches. (**b**) Coomassie-stained SDS-PAGE of eluates from Avicel affinity purifications of cleared *S*. *coelicolor* whole cell lysate from wild-type, ∆*filP*, and ∆*scy* strains. (**c**) Western blot analysis of the eluted proteins using polyclonal rabbit anti-FilP antibody or anti-DivIVA antiserum. (**d**) Coomassie-stained SDS-PAGE of Avicel affinity purification eluates from recombinant expressed FilP, Scy or DivIVA. OmpF band is indicated with an asterisk (*). (**e**) Graphical representation of coiled-coil domains predicted for FilP, Scy and DivIVA using COILS^36^. Coiled-coil domains are shown as black boxes. The number on the right side of each schematic corresponds to the protein length as the number of amino acids.

To evaluate whether cellulose affinity could be expected for FilP from *S*. *coelicolor*, the protein sequences of the two orthologue proteins FilP and AbpS were aligned using Clustal Omega (Fig. 1a). The sequence identity was determined to be >93%. Next, the identity of FilP orthologues from 16 additional species of *Streptomyces* showed that the identity ranged from 88 to 100% (Supplementary Table S1).

Experimental support for cellulose affinity of FilP orthologues was obtained by performing Avicel affinity purification. Whole cell lysate from *S*. *coelicolor* was incubated with Avicel, washed with 1 M NaCl, and eluted with 6 M urea, as described for AbpS^18^. Proteins eluted from the Avicel were separated by polyacrylamide gel electrophoresis (PAGE). Two major Coomassie-stained bands were detected, one corresponding to the expected size of FilP (SCO5396, Q9K4B5) and a larger band that were identified by mass spectrometry as Scy (SCO5397, Q9L2C3). Avicel affinity purification of the lysate from the *S*. *coelicolor* strains with ∆*filP* and ∆*scy* deletions confirmed that there was a direct cellulose interaction (Fig. 1b). Furthermore, the widespread property of cellulose affinity of FilP and Scy orthologues was confirmed in three additional species: *S*. *lividans*, *S*. *kanamyceticus*, and *S*. *venezuelae* (Supplementary Fig. S1).

DivIVA could be detected in all Avicel affinity eluates using western blot, suggesting that it displays cellulose affinity, although it could not be detected by Coomassie-staining (Fig. 1c). Avicel affinity purification of recombinant FilP, Scy and DivIVA expressed in *E*. *coli* verified that there was a direct protein-cellulose interaction and that cellulose binding was not dependent on *Streptomyces*-specific posttranslational modifications (Fig. 1d).

There is little sequence identity between FilP, Scy and DivIVA, and no previously described cellulose-binding domain could be detected. The most pronounced feature shared by FilP, Scy and DivIVA is their coiled-coil domains (Fig. 1e). Recombinant expression and affinity purification yielded stable FilP and DivIVA, while some degradation could be observed for Scy. The background binding to Avicel was low in the whole cell lysate from both *Streptomyces* and *E*. *coli* (Fig. 1b,d). However, Avicel affinity purification from *E*. *coli* lysate yielded a band of approximately 40 kDa, which was identified by mass spectrometry as OmpF (C9E713). Taken together, these data suggest that cellulose affinity is an intrinsic property of FilP, Scy and DivIVA.

### Deletion of FilP or Scy does not affect the tip localisation of cellulose-like molecules

**Figure 2:**
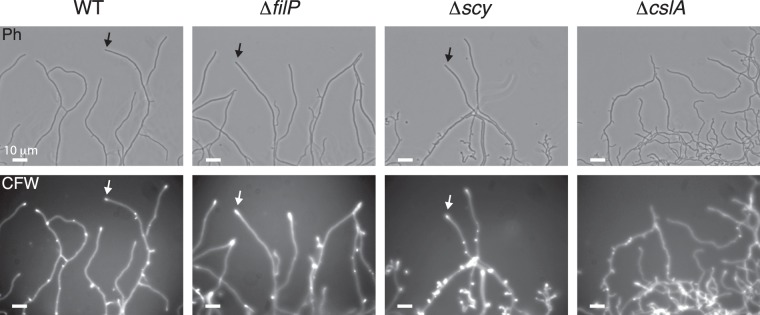
Calcofluor white staining of *S*. *coelicolor* hyphae. The strains were cultured on cellophane overlaid on TSA for 16 h. Calcofluor white staining revealed the cellulose-like molecules at the hyphal tips. Arrows indicate examples of hyphae with apically localised cellulose-like staining.

The effect of FilP and Scy on the localisation of the cellulose-like molecule was analysed by calcofluor white staining of hyphae from wild-type, ∆*filP*, ∆*scy* and ∆*cslA* strains of *S*. *coelicolor*. A cellulose signal was detected at the tip of wild-type hyphae but not in the ∆*cslA* strain. However, no effect was observed on the localisation of the cellulose-like molecule at the tip in ∆*filP* or ∆*scy* mutants (Fig. 2).

### Cellulose affinity of IF-like proteins and DivIVA are widespread and include metazoan IF

**Figure 3:**
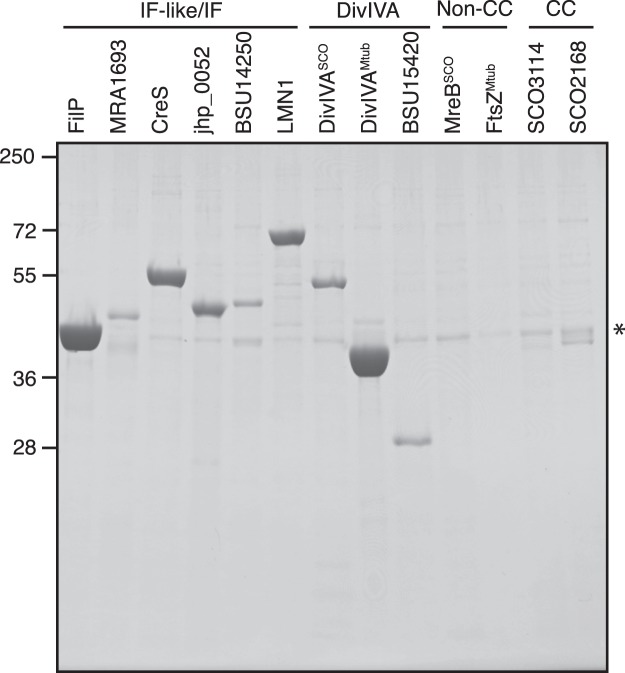
Cellulose affinity of IF-like, DivIVA and IF proteins. Coomassie-stained SDS-PAGE from Avicel affinity purification of recombinantly expressed proteins from *S*. *coelicolor* (SCO5396, Q9K4B5, FilP, predicted molecular weight (pM_w_) = 35 kDa), *M*. *tuberculosis* (MRA_1693, A5U340, pM_w_ = 35 kDa), *C. crescentus* (CCNA_03813, A0A0H3CD98, pM_w_ = 50 kDa), *H*. *pylori* (jhp_0052, Q9ZN08, pM_w_ = 38 kDa), *B*. *subtilis* (BSU14250, O31700, pM_w_ = 38 kDa), *C*. *elegans* (LMN1, Q21443, PM = 64 kDa), *S*. *coelicolor* DivIVA (SCO2077, Q9S2X4, pM_w_ = 41 kDa), *M*. *tuberculosis* DivIVA (wag31, A5U4H2, pM_w_ = 28 kDa) *B*. *subtilis* DivIVA (BSU15420, P71021, pM_w_ = 19 kDa), the non-coiled-coil (Non-CC) protein MreB (SCO2611, Q9L1G6, pM_w_ = 36 kDa) from *S*. *coelicolor* and FtsZ (ftsz, A5U4H7, pM_w_ = 39 kDa) from *M*. *tuberculosis*, the coiled-coil (CC) proteins (SCO3114, pM_w_ = 33 kDa and SCO2168, pM_w_ = 28 kDa) from *S*. *coelicolor*. The asterisk (*) indicates the Avicel binding protein OmpF from the *E*. *coli* lysate.

Coiled-coil IF-like proteins from *M*. *tuberculosis*, *H*. *pylori*, *C*. *crescentus*, *B*. *subtilis*, lamin from *C*. *elegans* and DivIVA from *B*. *subtilis* and *M*. *tuberculosis* were expressed in *E*. *coli*, and the cellulose affinity was examined by Avicel affinity purification. All tested IF-like proteins, DivIVA, and metazoan lamin displayed Avicel affinity (Fig. 3). Two additional coiled-coil proteins, SCO3114 and SCO2168, displayed weak affinity. This is consistent with the observation that only FilP and Scy are isolated as major bands from whole cell lysate of *S*. *coelicolor*. Two non-coiled-coil proteins, MreB and FtsZ, were used as controls and did not display any affinity to Avicel. The data presented demonstrate that IF-like proteins and DivIVA from different prokaryotes and the *bona fide* IF protein lamin display cellulose affinity.

### Characterisation of coiled-coil protein interaction with cellulose

**Figure 4:**
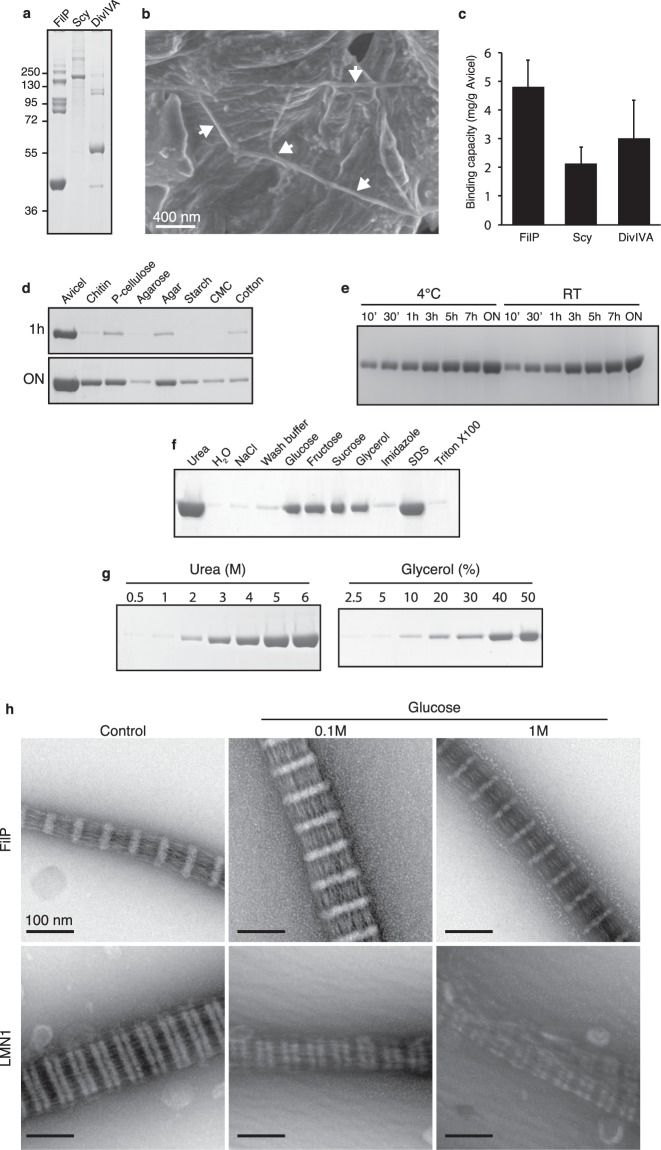
Characterisation of protein binding to Avicel. (**a**) Coomassie-stained SDS-PAGE of FilP, Scy and DivIVA treated with DSP while bound to Avicel. (**b**) FilP filament on the surface of an Avicel particle indicated by white arrows visualised by SEM. (**c**) FilP, Scy and DivIVA binding capacity to Avicel in whole cell lysate. Measurements of binding capacity were made in triplicates in three independent experiments. Error bars indicating standard deviations are shown. (**d**) Coomassie-stained SDS-PAGE of FilP binding to Avicel, chitin, phosphate cellulose, agarose, agar, starch, carboxymethyl cellulose (CMC), and cotton at 1 h and overnight. (**e**) Coomassie-stained SDS-PAGE showing FilP binding over time at 4 °C and room temperature. (**f**) Desorption of Avicel bound FilP by different chemicals (6 M urea, MQ water, 2 M NaCl, wash buffer, 40% glucose, 40% fructose, 40% sucrose, 40% glycerol, 2 M imidazole, 1% SDS, 1% Triton X-100) visualised by Coomassie-stained SDS-PAGE. (**g**) Coomassie-stained SDS-PAGE showing elution-potential of buffers containing different concentrations of urea or glycerol. (**h**) TEM images of negatively stained FilP and lamin in presence of 0.1/1 M glucose. Full-length gels are presented in Supplementary Fig. S2.

Oligomerisation of coiled-coil proteins generally leads to the exposure of hydrophilic amino acids on the surface and the embedding of hydrophobic amino acids. The oligomerised state of Avicel-bound protein was therefore investigated. Whole cell lysates from *E*. *coli* containing FilP, Scy or DivIVA were incubated with Avicel, washed, and crosslinked before elution. The Coomassie-stained gel showed several larger bands for all three proteins representing oligomerised proteins, which suggests that oligomerised protein bound cellulose (Fig. 4a). Accordingly, SEM visualisation of FilP incubated with Avicel showed filaments on the surface of the cellulose particles (Fig. 4b).

Next, the Avicel binding capacity of FilP, Scy and DivIVA was quantified using the whole cell lysate of *E*. *coli*. The cellulose particles were saturated with lysate containing target protein. The purity of eluted protein was verified by PAGE before the protein concentration was measured. The binding capacity in whole cell lysate was 4.8 (±0.9) mg/g Avicel for FilP, 2.1 (±0.6) mg/g Avicel for Scy and 3.0 (±1.3) mg/g Avicel for DivIVA (Fig. 4c).

FilP was selected for further studies of cellulose affinity by coiled-coil proteins due to its stability, binding capacity, and the opportunity for comparison with results from the FilP orthologue AbpS. First, the specificity of the cellulose interaction was tested by comparing FilP binding to different types of cellulose and polysaccharides (Fig. 4d). FilP interaction with crystalline cellulose was observed to be more efficient at both one hour and after overnight incubation compared to chitin, phosphate cellulose, agar and cotton while there was no binding to agarose, starch or carboxymethyl cellulose. The FilP binding to Avicel was investigated in a time course experiment (Fig. 4e). The binding increased with time and 50% of the protein could be recovered after 30 min when compared to overnight incubation. The binding of FilP to Avicel did not differ between 4 °C and room temperature (Supplementary Fig. S2a).

FilP, Scy and DivIVA had so far been eluted by the addition of buffer containing 6 M urea leading to the denaturation of bound proteins. Cellulose is composed of β(1 → 4) linked D-glucose units, and cellulose-binding proteins must therefore interact with these glucose units. The eluting potential of buffer containing sugars and other compounds was therefore tested. Glucose, fructose, sucrose, and the small sugar alcohol glycerol eluted FilP with comparable efficiency (Fig. 4f). The heterocyclic molecule imidazole was used as a control for cyclic compounds such as sugars and did not elute FilP.

The exclusive desorption of FilP by sugars and polyols was confirmed by testing additional sugars, polyols, and salts (Supplementary Table S2). The desorption of cellulose-binding proteins using water has been reported^23,24^. However, FilP binding to cellulose was not disrupted by water. Additionally, the FilP-cellulose interaction tolerated high salt concentrations (2 M NaCl) and changes in pH ranging from pH 3 to 10. The anionic denaturing agent SDS had the same eluting potential as 6 M urea, while the non-ionic detergents Triton X-100 and Tween-20 did not disrupt the cellulose interaction. Similar results were obtained for Scy (Supplementary Fig. S2b). However, DivIVA was not as efficiently eluted by sugars or polyols (Supplementary Fig. S2b). The efficiency of desorption was evaluated for urea and glycerol. A decrease in the concentration of urea to 5 M eluted >80% protein compared to 6 M urea. The same effect was observed when the glycerol concentration was reduced from 50% to 40% (Fig. 4g).

FilP and lamin both formed striated filaments *in vitro* and displayed cellulose affinity, even though they share less than 25% sequence identity. Glucose had no observable effect on either FilP or lamin filamentation, although FilP could be desorbed from Avicel by the addition of glucose as a competitive ligand (Fig. 4h). These results show that oligomerised coiled-coil proteins interacted with cellulose and that FilP bound to cellulose can be eluted by denaturation or by sugars and small polyols to obtain a non-denatured protein.

### Isolation of natively expressed non-tagged coiled-coil proteins using Avicel

**Figure 5:**
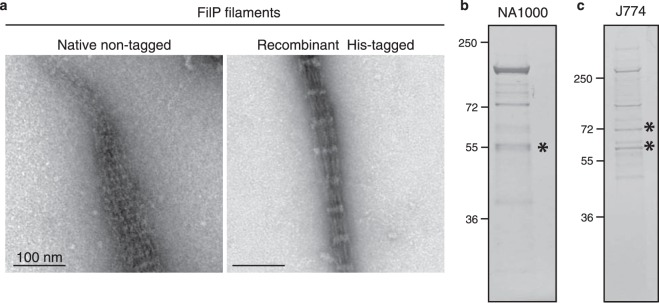
Isolation of IF-like proteins from endogenous lysates. (**a**) TEM images of negatively stained filaments formed by natively expressed FilP and recombinant His-tagged FilP. (**b**) Coomassie-stained SDS-PAGE of eluates from Avicel affinity purification of *C*. *crescentus* NA1000 whole cell lysate. Gel band containing crescentin is indicated with an asterisk (*). (**c**) Coomassie stained SDS-PAGE of eluates from Avicel affinity purification of mouse cell J774 whole cell lysate. Gel bands containing IF proteins are indicated with an asterisk (*).

The strong affinity to cellulose by FilP enabled purification of natively expressed non-tagged FilP from the *S*. *coelicolor* deletion strain ∆*scy* and allowed the study of filamentation *in vitro*. The filaments formed *in vitro* showed a characteristic striated pattern (Fig. 5a). These *in vitro* filament structures are similar to filaments formed by recombinantly expressed protein^1,10^.

The successful isolation of FilP from *S*. *coelicolor* lysate and the observed cellulose affinity of recombinant IF-like proteins from other species suggest that Avicel affinity purification could be used to isolate natively expressed IF-like proteins from other species. Hence, Avicel affinity purification was performed on whole cell lysate from *C*. *crescentus* (NA1000), which isolated crescentin (CreS, A0A0H3CD98) (Fig. 5b). However, there were other Avicel binding proteins in the *C*. *crescentus* lysate. The additional bands from *C*. *crescentus* were identified by mass spectrometry to be TonB outer membrane proteins (CCNA_03108, CCNA_00210, CCNA_02277, CCNA_01826). However, the absence of coiled-coil domains in TonB suggests a non-coiled-coil dependent interaction. TonB proteins have previously been reported to be involved in sensing environmental cellobiose^25^.

Next, Avicel affinity purification was performed on a cell lysate from the mouse macrophage cell line J774, which resulted in four major binding proteins (Fig. 5c). Identification by mass spectrometry revealed that the *bona fide* IF proteins lamin (P48678) and vimentin (P20152) were successfully isolated. The two additional proteins were identified as Plectin (Q9QXS1) and Splicing factor Spfq (Q8VIJ6). Both Plectin and Spfq contain coiled-coil domains. Hence, it is possible that these domains mediate the binding to cellulose. Thus, cellulose affinity can be utilised to isolate IF-like and IF proteins from whole cell lysates, and as shown for FilP, it could allow for *in vitro* studies of natively expressed non-tagged protein.

### Avicel binding is mediated by coiled-coil domains

**Figure 6:**
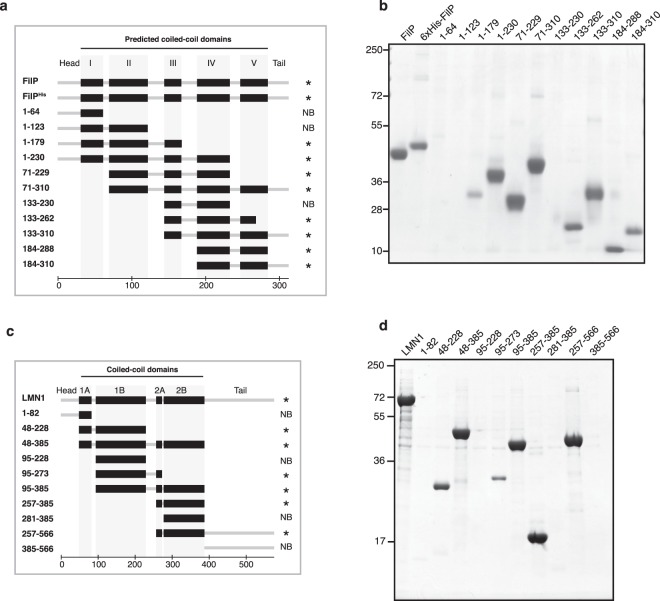
Cellulose-binding domains of FilP and lamin. (**a**) Graphical representation of FilP constructs tested for cellulose affinity. Predicted coiled-coil domains are shown as black boxes. Constructs are marked with an asterisk (*) if interacting with cellulose or non-binding (NB). The scale bar at the bottom refers to amino acid residues. (**b**) Coomassie-stained SDS-PAGE of Avicel affinity purification of FilP constructs. (**c**) Graphical representation of lamin constructs tested for cellulose affinity. Predicted coiled-coil domains are shown as black boxes. Constructs are marked with an asterisk (*) if interacting with cellulose or non-binding (NB). The scale bar at the bottom refers to amino acid residues. (**d**) Eluates from Coomassie-stained SDS-PAGE of Avicel affinity purification of lamin constructs.

FilP is composed of a head and tail domain and five predicted coiled-coil domains (domains I-V). Fragments were constructed around these coiled-coil domains, expressed in *E*. *coli*, and tested for their ability to bind Avicel in whole cell lysate (Fig. 6a,b). Efficient cellulose affinity was detected for the constructs containing a combination of domains II and IV (e.g. fragment 71–229) or IV and V (e.g. fragment 184–288). A construct containing domains III, IV, and a truncated version of domain V (fragment 133–262) was still able to bind cellulose and a weaker affinity was observed for the construct containing domains I, II, and III (fragment 1–179). Interestingly, the alignment of FilP from 17 species of *Streptomyces* showed a higher degree of sequence conservation at the C-terminal part containing coiled-coil domains IV and V (Supplementary Fig. S3a). Furthermore, cellulose affinity was observed for both an N-terminal and a C-terminal fragment of Scy, suggesting that it had more than one binding domain, as observed for FilP (Supplementary Fig. S3b).

The importance of coiled-coil domains for cellulose binding in the metazoan IF protein lamin was investigated by designing fragments around the coiled-coil domains 1A, 1B, 2A, and 2B (Fig. 6c,d). Lamin fragments containing the intact rod domain but lacking the head and tail domains bound to Avicel (fragment 48–385) and fragments consisting of domain 1A (fragment 1–82), 1B (fragment 95–228) or 2B (fragment 281–385) alone did not bind cellulose. However, binding was detected for constructs containing domains 1A and 1B (fragment 48–228), 1B and 2A (fragment 95–273), and domains 2A and 2B (fragment 257–385). These results suggest that the binding of lamin to cellulose depends on the combination of coiled-coil domains, as observed for FilP.

### Function of Avicel-binding coiled-coil domains as a protein tag for protein purification

**Figure 7:**
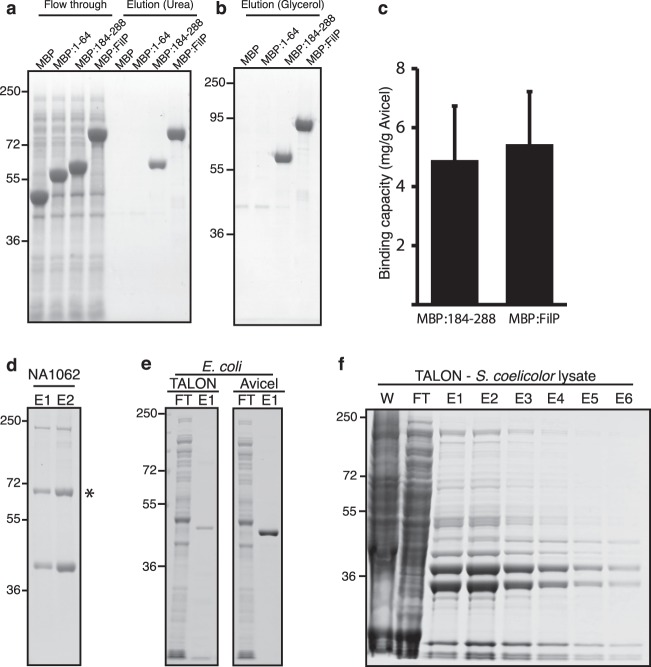
Utilisation of Avicel interacting coiled-coil domains for isolation of fusion proteins. Coomassie-stained SDS-PAGE of MBP-FilP constructs eluted by (**a**) 6 M urea and (**b**) 40% glycerol. (**c**) Avicel binding capacity of MBP:184–288 and MBP:FilP in whole cell lysate eluted by denaturation. Measurements of binding capacity were made in triplicates in three independent experiments. Error bars indicating standard deviations are shown. (**d**) Avicel affinity purification of lysate from merodiploid NA1062 expressing FilP-YPet, FilP and Scy. The band corresponding to the size of a FilP-YPet fusion is highlighted by an asterisk (*). (**e**) Batch purification of His-tagged FilP from *E*. *coli* lysate using TALON and Avicel. (**f**) TALON batch purification of His-tagged FilP expressed in *S*. *coelicolor* ∆*filP* from cleared whole cell lysate (W = whole lysate, FT = flow through, E = elution 1–6).

Next, we investigated the potential of using Avicel affinity purification to isolate target proteins fused to Avicel binding FilP constructs. Avicel affinity purification showed that neither maltose binding protein (MBP) alone nor MBP fusions with coiled-coil domain I (fragment 1–64) were able to bind cellulose. However, fusions of MBP with fragment 184–288 or full length FilP were successfully isolated (Fig. 7a). Elution of MBP-fusions by 40% glycerol was possible, as observed for recombinant FilP (Fig. 7b). NusA fusion proteins were also constructed and yielded similar results (Supplementary Fig. S4).

The binding capacities of MBP:FilP and MBP:184–288 were 5.4 (±1.4) and 4,9 (±1,7) mg/g Avicel, respectively (Fig. 7c). Avicel affinity purification using lysate from the *S*. *coelicolor* strain NA1062 expressing a FilP-YPet fusion protein yielded a band with the predicted size of the FilP-fusion protein (Fig. 7d). This demonstrates that FilP-fusion proteins can be isolated from *Streptomyces* lysates. While the background binding of Avicel and TALON resin in *E*. *coli* lysate was comparatively low, the background binding of *Streptomyces* lysate to Avicel was substantially lower compared to TALON resin (Figs 1b and 7d–f). These results show a practical application of utilising the cellulose affinity of FilP for the isolation of tagged recombinant protein from *E*. *coli* and *Streptomyces*.

## Discussion

In this study, we have demonstrated cellulose binding by coiled-coil IF-like, DivIVA and *bona fide* IF proteins. The first observation of cellulose affinity was made for a FilP orthologue from *S*. *reticuli*^18^. We set out to determine the prevalence of cellulose affinity among FilP orthologues and observed that FilP from three additional *Streptomyces* species displayed cellulose affinity. The high degree of conservation across the genus suggests that the cellulose affinity might be a general property of FilP orthologues. Moreover, DivIVA and the IF-like protein Scy are also able to bind cellulose. DivIVA is not considered an IF-like protein but it is reported to share a similar coiled-coil domain organisation to that of FilP and Scy^15^. Although the genome of *S*. *reticuli* encodes a Scy and DivIVA orthologue, only the FilP orthologue AbpS was identified as binding cellulose in *S*. *reticuli*^18^. Quantifications of the FilP orthologue AbpS estimated that there is 5 µg AbpS/10 g of mycelia^26^, which suggests that Avicel affinity purification can be used to isolate proteins of low concentration.

It has been proposed that the cellulose-like fibrils at the tip of the *Streptomyces* hyphae could be anchored by a tip-localised cellulose binding protein^21^. However, no effects could be observed upon deletion of FilP or Scy, which suggests that FilP and Scy are not involved in anchoring the cellulose-like polymers at hyphal tips. It is however possible that the redundancy of cellulose affinity provided by FilP, Scy, and DivIVA requires the deletion of all three proteins to observe an effect. Attempts to create a *divIVA*-deleted strain have failed due to its essential function^14^. Hence, it cannot be ruled out that FilP, Scy and DivIVA are involved in the anchorage of the cellulose-like molecules at the tip.

Cellulose affinity is not limited to FilP, Scy and DivIVA from *Streptomyces*. Binding of coiled-coil IF-like and DivIVA protein from other prokaryotic species and the *bona fide* IF protein lamin demonstrate that cellulose affinity of certain coiled-coil proteins is widespread. The sequence identity to FilP for the tested IF-like and DivIVA proteins range from 21–29%. Interestingly, these IF-like proteins and DivIVA proteins interacted with Avicel despite low sequence identity, although with different efficiency. The identity to FilP for the coiled-coil proteins SCO2168 and SCO3114 were 28% and 33% respectively, and the identity for the non-coiled-coil cytoskeletal proteins FtsZ and MreB were both 22%. The binding of oligomerised FilP, Scy and DivIVA demonstrates that oligomerisation and cellulose binding are not mutually exclusive and suggests that the interaction with cellulose is mediated by the hydrophilic outer surface of the coiled-coils. The more efficient binding to crystalline cellulose suggests that the compact linear arrangement of the cellulose molecules is beneficial for the interaction. Higher binding affinity to crystalline cellulose compared to other types of polysaccharides was also reported for the FilP orthologue AbpS^18^. FilP was found to also bind chitin. Although chitin is not a type of cellulose, there are CBM proteins described that also show affinity for chitin^27^.

The desorption of cellulose-bound FilP was accomplished by denaturation or by a high concentration of competitive ligands such as sugars and small polyols. Proteins containing classical CBM have previously been reported to be desorbed by sugars and polyols^23,28^. Although it remains uncertain whether an actual coiled-coil-carbohydrate interaction occurs *in vivo*, competitive binding of sugars suggests that a candidate ligand does not necessarily have to be a large polysaccharide.

Avicel affinity purification allowed for the isolation of sufficient amounts of natively expressed FilP from *S*. *coelicolor* to study the *in vitro* filamentation. This shows promise for the isolation of other natively expressed cellulose-interacting proteins like Scy and DivIVA. Avicel affinity purification could also be applied for the isolation of natively expressed IF-like and IF proteins from other species, as demonstrated for *C*. *crescentus* and mouse cells. Further, this also suggest that cellulose affinity could be used as a biochemical assay when searching for IF-like proteins in various species. The background binding to Avicel in *Streptomyces spp*. and *E*. *coli* lysates is low. However, the higher background binding in lysates from e.g. *C*. *crescentus* and mouse cells must be considered before utilising Avicel protein isolation methods.

Certain coiled-coil domains of FilP and lamin mediate the interaction to cellulose. To our knowledge, the cellulose affinity of coiled-coil domains has not been reported in the literature to date. FilP and lamin share little sequence identity, and therefore, cellulose-binding activity cannot be ascribed to any common motif within the coiled-coil domains. The exact molecular interaction of how coiled-coil domains bind to cellulose remains to be determined.

The cellulose-binding activity of CBMs has previously been utilised as protein purification tags^24,28–33^. The use of cellulose as a matrix for protein purification has several advantages, including being inert, inexpensive and having low nonspecific binding^23^. Fusions of cellulose-binding coiled-coil domains derived from FilP to target-proteins allowed for their purification using Avicel affinity purification. A recent publication evaluated the applicability of multiple conventional protein tags for the analysis of regulatory pathways by protein-protein interaction in *Streptomyces*^34^. The potential for Avicel affinity-based purification as an additional tool for *Streptomyces* protein studies is underlined by the significantly lower background binding in the lysate of *S*. *coelicolor* by Avicel particles compared to TALON, and that a FilP-fusion protein was efficiently recovered from *Streptomyces*. Moreover, the release of Avicel-bound fusion proteins using a cryoprotectant such as glycerol could be beneficial for the isolation of certain proteins.

In this study, we report that the affinity to crystalline cellulose is intrinsic for IF and prokaryotic IF-like proteins. Certain coiled-coil domains were demonstrated to mediate the interaction with cellulose. The cellulose affinity displayed by the IF-like proteins allowed for the isolation of native and recombinantly expressed fusion proteins.

## Methods

### Strains

*Streptomyces coelicolor*, *Escherichia coli*, and other species used in this work are listed in Supplementary Table S3. *E*. *coli* strains were grown on LA plates or LB media containing ampicillin (100 μg/mL) or kanamycin (50 μg/mL) and isopropyl β-D-1-thiogalactopyranoside (IPTG) was added to induce protein expression. *S*. *coelicolor* strains were grown on SFM agar, TSA, or TSB at 30 °C^35^. Thiostrepton (5 μg/mL) was added when culturing NA1062. *Caulobacter crescentus* was grown in PYE at 30 °C.

### Construction of plasmids and recombinant expression

The plasmids used in this study are listed in Supplementary Table S4. DNA manipulation and cloning were carried out according to standard protocols. All constructs were verified by nucleic acid sequencing. The sequences of primers used for cloning are specified in Supplementary Table S5.

### Bioinformatic analysis

The coiled-coil domains of FilP, Scy and DivIVA were predicted using the COILS algorithm^36^. Sequence identities and alignments were determined using Clustal Omega.

### Avicel affinity purification and protein purification

*S*. *coelicolor* was grown in TSB, harvested after 48 h and disrupted by sonication at 4 °C in lysis buffer (0.1 M phosphate buffer pH 7.2, 0.15 M NaCl, 0.1% Triton X-100, 1x protease inhibitor cocktail). Lysates containing soluble proteins were cleared by centrifugation for 15 min at 20,000 g. Avicel affinity purification was performed as described by Walter *et al*.^18^. Cleared or non-cleared lysates were incubated with Avicel (15 mg/ml) in sonication buffer overnight (approximately 16 h) at 4 °C unless stated otherwise. The Avicel particles were washed three times by centrifugation at 700 g with wash buffer (50 mM sodium phosphate buffer pH 7.2, 1 M NaCl).

Crosslinking experiments were performed using 0.25 mM dithiobis[succinimidylpropionate] (DSP) for 10 min at room temperature. Lysis buffer containing 6 M urea was used to elute bound protein unless stated otherwise. Eluates were boiled in SDS sample buffer for 10 min before separation by SDS-polyacrylamide gel electrophoresis (PAGE) and staining with Coomassie brilliant blue. Protein purifications using TALON (Clontech) were performed according manufacturer’s instructions using the same buffer conditions used for Avicel affinity purification. Proteins were eluted using 150 mM imidazole in lysis buffer.

### Western blotting

Eluates were run on SDS PAGE gels, and FilP and DivIVA were detected by Western blotting using a primary polyclonal rabbit anti-FilP antibody or anti-DivIVA antiserum^37^. Secondary horseradish peroxidase-conjugated anti-rabbit was used for detection. Blots were visualised using ECL reagents. Rabbit antiserum raised against *S*. *coelicolor* FilP was purchased from Agisera AB, Sweden. FilP antibodies from the serum were purified against two fragments of FilP to increase the specificity: fragment 1–230 and fragment 133–310. The FilP fragments were expressed in BL21 and purified by His-tag affinity purification. Anti-FilP antibodies were purified according to the antibody purification protocol developed by Betts *et al*.^38^.

### Mass spectrometry preparation and analysis

Three independent Avicel affinity purifications aimed for mass spectrometry identification were performed to ensure reproducibility. Proteins separated by SDS-PAGE and stained with PageBlue and single bands were cut out and sent for identification to Linköping University, where general in-gel digestion was performed as described by Shevchenko *et al*.^39^. Briefly: stained protein bands were excised, destained, reduced by DTT, alkylated by iodoacetamide and digested by 0.005 µg/µL trypsin (Thermo scientific, Rockford, IL, USA) over night at 37 °C. Peptides were dried, dissolved in 0.1% formic acid and analysed by liquid chromatography mass spectrometry (LC-MS/MS). Peptides were separated by reverse phase chromatography on C18 columns with 5 µm particle size (20 mm × 100 µm pre-column followed by a 100 mm × 75 µm column) (NanoSeparations, Netherlands) at a flow rate 300 nL/min in a 55 min linear gradient from 0.1% formic acid/water to 0.1% formic acid/100% acetonitrile. Data acquisition was performed with LTQ Orbitrap Velos Pro hybrid mass spectrometer (Thermo Scientific). Data files were analysed by the Proteome Discoverer 1.4 (Thermo Scientific) and the search algorithm SEQUEST HT was used against the UniProt database of appropriate species. Trypsin and semi-tripsin were used as enzyme with one-missed cleavages allowed; methionine oxidation and carbamidomethylation of C, D, E, were set as variable modifications. The precursor mass tolerance was set to 10 ppm with 0.6 Da fragment mass tolerance. The result was filtered with peptide confidence value “high” and target false discovery rate 0.05. Proteins were identified with at least 2 unique peptides of rank 1 in top scored proteins with high confidence.

### Light microscopy

Cultures for phase contrast and fluorescence microscopy were grown on cellophane on TSA plates for 16 h at 30 °C. Calcofluor was added 10 minutes before viewing the samples. All light microscopy was performed using a Nikon Eclipse 90i fluorescence microscope equipped with appropriate filter sets, a Hamamatsu ORCA-ER camera, and NIS Elements AR software. Images were processed using Adobe Photoshop CC software.

### Electron microscopy

Purified FilP and lamin were eluted in 6 M urea and were therefore in a denatured state. Protein samples were dialysed against a filamentation buffer (50 mM Tris-HCl pH 6.8) for 30 min at 4 °C. The dialysed samples were pipetted onto a copper grid coated with formvar and carbon, prepared by glow discharge and negatively stained using 1.5% uranyl acetate. Samples were observed with a JEOL 1230 transmission electron microscope, and images were recorded with a Gatan Orius CCD camera. For scanning electron microscope (SEM) observations, Avicel was incubated with FilP for 30 min in filamentation buffer and added to glass slides that were coated with poly-L-lysine. The samples were critically point dried and coated with 1 nm of iridium before being analysed with a Merlin Field Emission SEM. Images were processed using Adobe Photoshop CC software.

## Electronic supplementary material

Supplementary information

## Data Availability

The authors declare that the data supporting the findings of this study are available within the article and its supplementary information files or from the corresponding authors on request.
