# Supplementary material for: Affinity to cellulose is a shared property among coiled-coil domains of intermediate filaments and prokaryotic intermediate filament-like proteins
Source: Sci Rep. 2018 Nov 8;8:16524. doi: 10.1038/s41598-018-34886-7 (PMC6224456; doi:10.1038/s41598-018-34886-7)
Supplement: Supplementary file 1 — Supplementary information [file 41598_2018_34886_MOESM1_ESM.pdf]

Affinity to cellulose is a shared property among coiled-coil domains of intermediate filaments and prokaryotic intermediate filament-like proteins

Niklas Söderholm[1], Ala Javadi[1], Isabel Sierra Flores[1], Klas Flärdh[2], Linda Sandblad[1*]

*Linda.sandblad@umu.se

[1]Department of Molecular Biology, Umeå University, 901 87 Umeå, Sweden

[2]Department of Biology, Lund University, 22362 Lund, Sweden

Supplementary information

Supplementary Table S1. Sequence identity of FilP orthologues.

Species	Identity to S. coelicolor FilP (%)
S. albus	95
S. anulatus	91
S. antibioticus	95
S. avermitilis	95
S. bingchenggensis	91
S. cattleya	88
S. clavuligerus	91
S. fradiae	88
S. griseus subsp. griseus	91
S. hygroscopicus subsp. jinggangensis	95
S. lividans	100
S. noursei	90
S. rapamycinicus	90
S. reticuli	90
S. roseosporus	91
S. scabiei	95
S. venezuelae	90

Supplementary Table S2. Desorption potential of FilP bound to Avicel by different compounds.

Sugars	Elutes	Concentration
Arabinose	Yes	2M
Fructose	Yes	2M
Galactose	Yes	2M
Glucose	Yes	2M
Glucosamine	Yes	2M
Maltose	Yes	2M
Mannose	Yes	2M
Sorbose	Yes	2M
Sucrose	Yes	2M
Xylose	Yes	2M
**Polyols**		
Arabitol	Weak	2M
Glycerol	Yes	2M
Sorbitol	Yes	2M
Mannitol	Weak	2M
Xylitol	Yes	2M
**Other**		
Bicine	No	2M
Ethanolamine	Yes	2M
Tricine	Yes	2M
EDTA	No	2M
Glycine	No	2M
Ethylene glycol	Yes	100%
Triton X-100	No	1%
Tween-20	No	1%
DMSO	No	40%
Na_2CO_3	No	2M
NaCl	No	2M
Na_2HPO_4	No	2M
$(NH_2)_4SO_4$	No	2M
KNO_3	No	2M
KCl	No	2M
$MgCl_2$	No	2M
$CaCl_2$	No	2M

Supplementary Table S3. Strains used in this study.

Bacterial strains	Strain/Description	Source
Streptomyces	WT M145	
	M145 ΔfilP::FRT (NA883)	(10)
	M145 ΔSCO5367::[aac(3)IV oriT] (NA336)	(10)
	M145 sco2836::Tn5062	(21)
	M145 ΔfilP::filP-ypet attPΦC31pNA937(tipAp-filP)	(10)
E. coli	DH5α	
	BL21 (DE3)	
C. caulobacter	NA1000	
B. subtilis	strain 168	
H. pylori	J99	
M. tuberculosis	H37Rv	
**Other**		
Mouse	J774 Macrophage cell line	

Supplementary Table S4. Plasmids used in this study.

Plasmid	Insert/ Description	Source
pETM13*	non-tagged FilP	This study
pET28a	N-terminally His-tagged FilP	(1)
pET28a	SCO5397 (Scy)	This study
pNIC28-Bsa4 LIC	Scy 1-1647	This study
pNIC28-Bsa4 LIC	Scy 1630-3981	This study
pET28a	SCO2077 (DivIVa)	This study
	**FilP fragments**	
pETM11*	1-64	This study
	1-123	This study
	1-176	This study
	1-230	This study
	133-310	This study
	71-229	This study
	71-310	This study
	133-230	This study
	133-262	This study
	133-210	This study
	184-288	This study
	184-310	This study
	**Fusions**	
pETM40*	Empty (MBP)	*
	MBP 1-64	This study
	MBP 184-288	This study
	MBP FilP	This study
pETM60*	Empty (NusA)	*
	NusA 1-64	This study
	NusA 184-288	This study
	NusA FilP	This study
	**IF/IF-like and DivIVa**	
pET28a	CreS	(6)
	JHP_0052	This study
pETM11*	MRA1693	This study
	BSU14520	This study
	Wag31 DivIVa	This study
	BSU15420 DivIVa	This study
pET21a	LMN1	**
	LMN1 fragments	
pET28a	LMN1 1-82	This study
	LMN1 48-228	This study
	LMN1 48-385	This study
	LMN1 95-228	This study
	LMN1 95-385	This study
	LMN1 257-566	This study
	**Other**	
pETM11*	MRA_2165 (FtsZ)	This study
	SCO3114 (coiled-coil protein)	This study
	SCO2168 (MreB)	This study
pET28a	SCO2611 (coiled-coil protein)	This study

(*) G. Steir, EMBL

(**) kindly provided by Prof. Krohne, Würzburg University

Supplementary Table S5. Primers used in this study.

Name	Description	Primer sequence
	**FilP constructs and fusion tags**	
OLS54	FilP-1-fwd	ATTTTCCATGGGCAGCGACACTTCCCCCTACG
OLS57	FilP-310-rev	ATATAGAATTCAGCGGGACTGCTGGGCCGGGA
OLS60	FilP-64-rev	ATCGCGAAT TCACTCTGCGTCGTTGACCTGGG
OLS61	FlP-71-fwd	ATTTTCCATGGGCGCCCGGGTCGAGAAGATC
OLS62	FilP-123-rev	ATATAGAATTCAGGCCTTGCGCTCGGCGGCGTA
OLS64	FilP-176-rev	ATATAGAATTCAGAAGTCGGCGGCGGCCTGCG
OLS66	FilP-230-rev	ATATAGAATTCAGGTCTGGCGGGCCCGGCG
OLS63	FilP-133-fwd	ATTTTCCATGGTCGAGAAGGCCAAGGGCGACGCC
OLS155	FilP-262-rev	ATATAGGTACCTCAGGCGGCCAGCTCGCG
OLS65	FilP-184-fwd	ATTTTCCATGGTCCGCGAGCAGTCGGAGCG
OLS68	FilP-288-rev	ATATAGAATTCACGCGGCGCCCGTGAGCGA
	**Scy**	
OLS183	Scy-1-fwd	ATATACATATGGTGCGGGGCTACGAGAGCC
OLS184	Scy-1326-rev	ATATAGAATTCCTAGCCGTCTGACGACTTGCC
OLS95	scy-1-fwd	TACTTCCAATCCATGCGGGGCTACGAGAGCCAG
OLS96	scy-1660-rev	TATCCACCTTTACTGGAGCTCCTCCACCCGCT
OLS97	scy-1630-fwd	TACTTCCAATCCATGCGGGTGGAGGAGCTCCAG
OLS98	scy-3981-rev	TATCCACCTTTACTGCTAGCCGTCTGACGACTTGCC
	**DivIVa**	
OLS178	SCO2077 DivIVa	ATATACATATGCCGTTGACCCCCG
OLS179	SCO2077 DivIVa	ATATAGAATTCTCAGTTGTCGTCCTCGTCGATC
	**IF-like/DivIVa/other**	
OLS84	MRA1693_1_fwd	CCTTCCATGGCATTGCCTCAACGCCCAAAC
OLS85	MRA1693_end_rev	GCGCGAATTCAAATGGCCCGTAGCTCAGC
OLS187	jhp_0052_1_fwd	ATATACATATGGGAACGCTCATTGAAAAATG
OLS188	jhp_0052_end_rev	ATATAGAATTCTTATTGTGGTTTTTGTGGTTGC
OLS195	BSU14250_1_fwd	ATATACCATGGCCAAAAGATCAGGTCC
OLS196	BSU14250_end_rev	ATATAGAATTCTTATTGAGGTTTTTTCTGTG
OLS082	Wag31 _1_rev	AATACCATGGCACCGCTTACACCTGCCGA
OLS083	Wag31 _end_rev	CGCGGAATTCAGTTTTTGCCCCGGTTGAA
OLS197	BSU15420_1_fwd	ATATACCATGGCCCCATTAACGCCAAATG
OLS198	BSU15420_end_rev	ATATAGGATCCTTATTCCTTTTCCTCAAATAC
OLS202	SCO2611_1_F_NdeI	ATATACATATGGGGAACTCAATGTCGTTC
OLS203	SCO2611_end_R_EcoRI	ATATAGAATTCTCATCTACGGGGCGAGG
OLS206	SCO3114_1_fwd	ATATACCATGGGCATGAGCGGTGCATCGGCGTC
OLS207	SCO3114_end_rev	ATATAGAATTCTCATTCCACCTCCACCGCAC
OLS204	SCO2168_1_fwd	ATATACCATGGGCATGATCTTCCGCGCGAAGG
OLS205	SCO2168_end_rev	ATATAGAATTCCTACTGCTTGTCGAAGCGCG
OLS088	FtsZ_1_fwd	TATTCCATGGCAACCCCACCGCAYAACTACYT
OLS089	FtsZ_end_rev	AGTTCTCGAGTCARCGGCGCATGAATGGC
	**LMN1**	
OLS224	LMN1_1_F	ATATACATATGTCATCTCGTAAAGGTACTCG
OLS225	LMN1_82_rev	ATATACTCGAGCTAAACTTCGATGTCGCGAATTTG
OLS226	LMN1_48_F	ATATACATATGCATTTGACTTCACTCAACAGTCG
OLS227	LMN1_228_R	ATATACTCGAGCTATTCTCCCTTGTGCTGTTGAAG
OLS228	LMN1_385_R	ATATACTCGAGCTACTCCTCCTCACCCTCAAGGAG
OLS229	LMN1_95_F	ATATACATATGTTCGAGGCGGAAAAGGCTCG
OLS230	LMN1_257_F	ATATACATATGGATCAAATCGAAGAGATGCG
OLS231	LMN1_566_R	ATATACTCGAGTTACATGATGGAACAACGATC

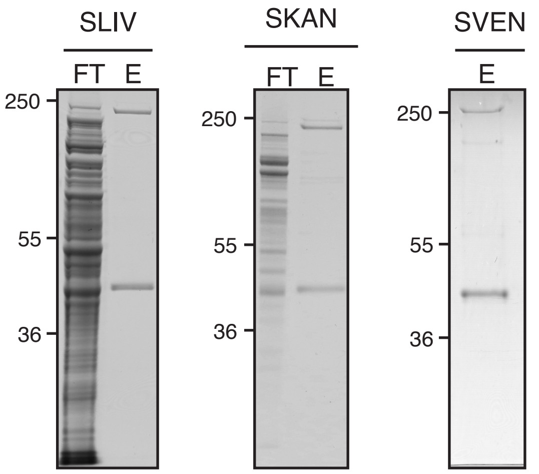

Figure S1. Cellulose binding protein from other species of Streptomyces. Coomassie-stained SDS-PAGE of eluates from Avicel affinity purifications of cleared whole cell lysate from *S. lividans* (SLIV), *S. kanamyceticus* (SKAN),*S. venezuelae* (SVEN). FT=flow through, E=elution.

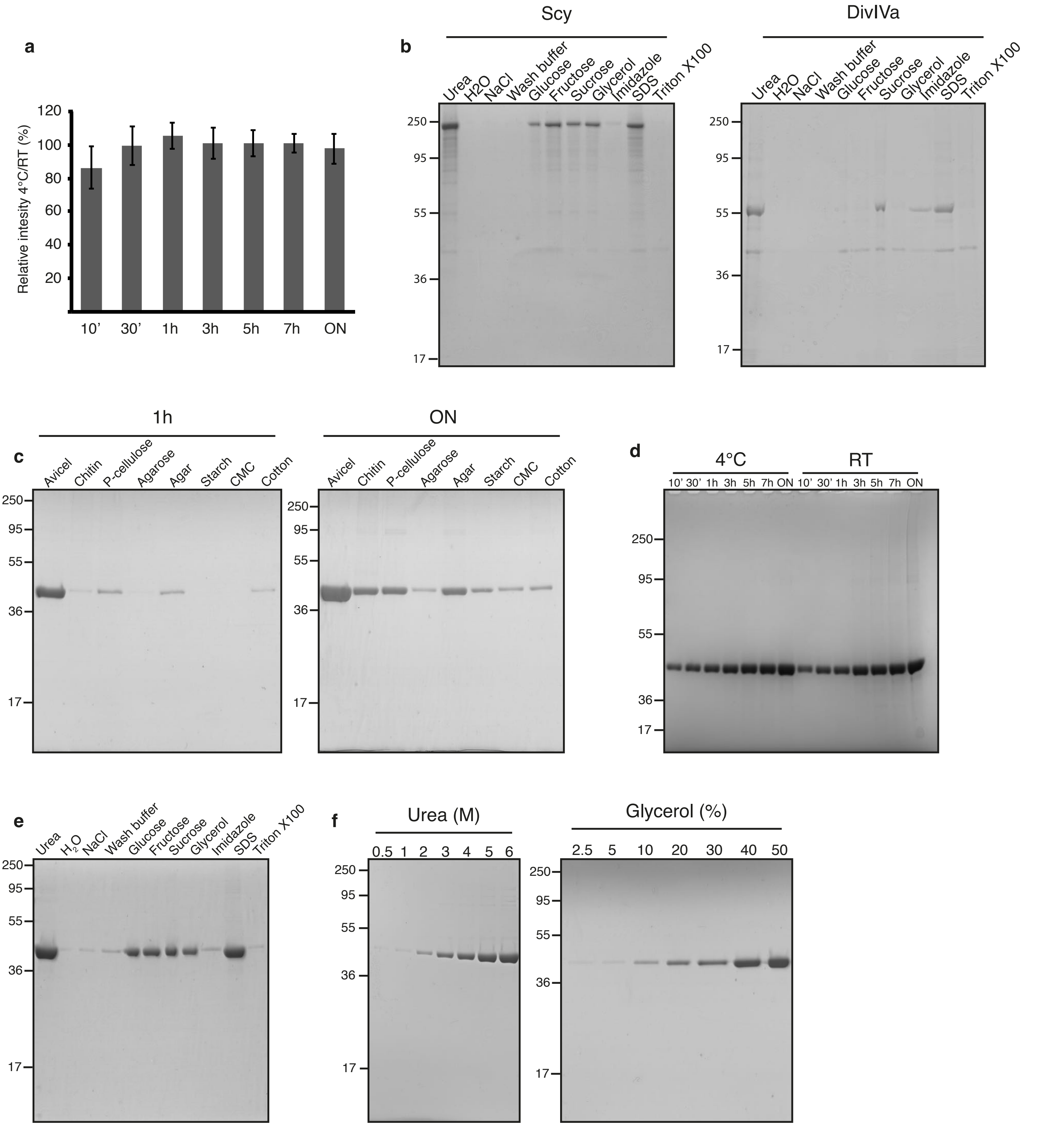

Figure S2. Characterisation of Avicel binding protein. (**a**) Quantification of FilP gel bands at given time points at 4°C and room temperature. The bars represent the relative intensity between the two conditions at each time point. Quantifications were made from three independent experiments. Error bars indicating standard deviations are shown. Coomassie-stained SDS-PAGE gels of (**b**) Scy and DivIVA, eluted from Avicel using buffers with supplements (6 M urea, MQ water, 2 M NaCl, Avicel assay wash buffer, 40% glucose, 40% fructose, 40% sucrose, 40% glycerol, 2 M imidazole, 1% SDS, 1% Triton X-100). (**c-f**) Full-length gels of cropped views from Fig. 4. (**c**) Uncropped view of Figure 4d. Coomassie-stained SDS-PAGE of FilP binding to Avicel, chitin, phosphate cellulose, agarose,agar, starch, carboxymethyl cellulose (CMC), and cotton at 1 h and overnight. (**d**) Uncropped view of Fig. 4e. Coomassie-stained SDS-PAGE showing binding over time at 4°C and room temperature. (**e**) Uncropped view of Fig. 4f. Desorption of Avicel bound FilP by different chemicals (6M urea, MQ water, 2M NaCl, wash buffer, 40% glucose, 40% fructose, 40% sucrose, 40% glycerol, 2M imidazole, 1% SDS, 1% Triton X-100) visualised by Coomassie-stained SDS-PAGE. (**f**) Uncropped view of Fig. 4g. Elution potential of buffer containing different concentrations of Urea or glycerol.

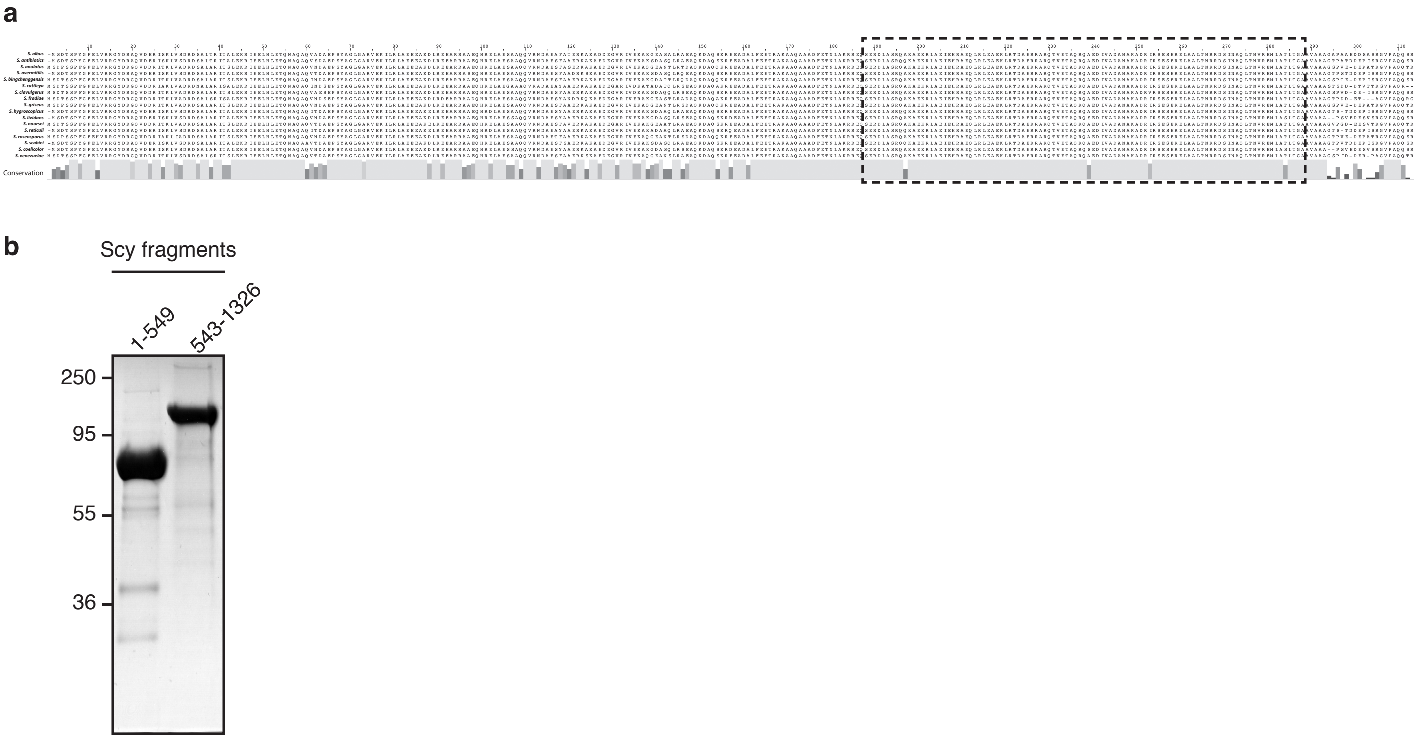

Figure S3. Cellulose binding domains. (**a**) Clustal Omega Alignment of FilP orthologues from 17 species of Streptomyces. Dotted black box highlight coiled-coil domains IV and V (amino acids 184-288). (**b**) Coomassie-stained SDS-PAGE of Avicel affinity purifications for binding-analysis of the N-terminal (1-549) and C-terminal (543-1326) Scy fragments.

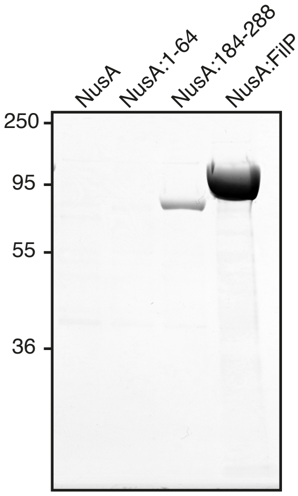

Figure S4. Utilisation of Avicel interacting coiled-coil domains for isolation of fusion proteins. Eluates from Avicel affinity purification of NusA-FilP constructs analysed by Coomassie-stained SDS-PAGE.
